# Reported Barriers and Enablers to Undertaking Childhood Vaccinations by Bangladeshi Parents in East London

**DOI:** 10.1093/eurpub/ckac129.107

**Published:** 2022-10-25

**Authors:** I Ali

**Affiliations:** LSHTM, London, UK

## Abstract

**Introduction:**

The Bangladeshi community living in the East London borough of Tower Hamlets is one of the UK's most socioeconomically deprived communities. Despite being a highly disadvantaged ethnic group with suboptimal health, the data suggests the uptake of several childhood vaccinations including the MMR vaccine is notably higher amongst this group, relative to other ethnic groups in Tower Hamlets.

**Methods:**

This study employs a qualitative research design. One-to-one, semi-structured interviews will be conducted with Bangladeshi parents, alongside relevant healthcare and public health professionals involved in vaccination delivery in Tower Hamlets to understand the barriers and enablers to childhood vaccinations. Interviews will be conducted in English by the researcher or in Bengali/Sylheti using an interpreter. Interviews will be audio-recorded, transcribed, translated and analysed using a thematic analysis. The socioecological model will be utilised as a theoretical framework to guide the data collection and analysis.

**Results and discussion:**

The preliminary results indicate parental trust in the safety of vaccinations, perceived health importance of childhood vaccinations, ease in accessibility and positive attitudes towards vaccinations within the community are notable enablers. Regarding barriers, parents have expressed reluctance on religious grounds towards childhood vaccinations which contain animal derivatives. The data also suggests differences exist between immigrant and non-immigrant parents in the decision-making process to undertake the vaccinations, with non-immigrant parents demonstrating a higher level of agency in their decision-making.

**Conclusions:**

The study provides valuable insight into the barriers and enablers for childhood vaccinations amongst the Bangladeshi community in Tower Hamlets. This data may inform tailored initiatives to improve childhood vaccination uptake amongst other underserved communities with suboptimal uptake.

